# Treatment of gingival recession defects using non-invasive pinhole technique with propolis application, a case report

**DOI:** 10.1016/j.ijscr.2021.106042

**Published:** 2021-05-26

**Authors:** Diana Mostafa, Obada Amer Mandil

**Affiliations:** a Periodontology and Oral Medicine Department. Alexandria University, Faculty of Dentistry, Egypt; b Preventive Dental Sciences, Vision Colleges, Riyadh, Saudi Arabia

**Keywords:** Gingival recession, Pinhole surgical technique, Root coverage, Propolis

## Abstract

**Introduction and importance:**

Despite the considerable surgical techniques that have been done for the root coverage, surgical difficulties, time, and patients' discomfort are still the main obstacles. However, the morbidity associated with the secondary graft sites has generated interest in new modalities to achieve the esthetic and functional requirements without complications, to reach patient comfort and satisfaction. In our study, we used a recent novel surgical technique which is called the pinhole surgical technique as it is a minimally invasive treatment that reverses gingival recession without using donor graft, flap elevation, or sutures. In this study, we also used propolis for root conditioning as it is a natural anti-infective, anti-inflammatory, and anti-oxidant agent.

**Presentation of the case:**

A 58-year-old systemically healthy female patient was referred to our periodontal clinics for the root coverage of the upper left canine and the first premolar which were diagnosed as Class II and Class I Miller's classification respectively. A pinhole surgical technique was done using propolis for root conditioning. A pinhole was created and the gingiva was pushed downwards until reaching the desired position coronally with the aid of collagen strips. Then, propolis was applied again postoperatively to enhance healing.

**Clinical discussion:**

Pinhole surgical technique can immediately cover exposed roots without incisions, donor site or flap reflection. In addition, the use of propolis in root conditioning showed positive results. This is due to its antioxidant and anti-inflammatory effects.

**Conclusion:**

Pinhole surgical technique using propolis is a promising modality that reaches the periodontist ambition for gingival recession defects.

## Highlights

• Pinhole surgical technique is considered as an effective alternative treatment for root coverage without the need for incisions, scalpel and donor site.
• In our case report, propolis was used as a natural antioxidant and antibacterial material in a novel protocol for root conditioning during root coverage procedure resulting postive outcomes.
• Topical application of propolis can be added to any oral surgical protocol to enhance healing capacity and minimize postoperative complications.

## 1 Introduction

Periodontal plastic surgeries are performed to prevent or treat anatomical, developmental, traumatic and plaque-induced defects of the gingiva, alveolar mucosa, and bone [1]. The main characteristic of gingival recession (GR) is the apical migration of marginal gingiva, displaced away from the cementoenamel junction (CEJ), exposing the root surface to the oral environment. Also, it is found in nearly all populations worldwide and is usually present with buccal surfaces of either single or multiple teeth [2].

Several techniques have been used to treat GR defects, including the creation of free gingival grafts, laterally positioned flaps or semilunar coronally positioned flaps, as well as guided tissue regeneration and connective tissue grafting [3]. Despite the considerable number of studies that have been done for root coverage (RC), surgical difficulties, time, and patients' discomfort are still the main obstacles. However, the morbidity associated with the secondary graft sites has sparked interest in and a need for other modalities to meet the esthetic and functional requirements without complications, while ensuring the patient comfort and satisfaction.

In our study, we used a recent non-invasive surgical technique which is called pinhole surgical technique (PST) as it reverses GR without using donor graft, flap elevation, or sutures [4]. This technique was introduced by John Chao [4] in 2012 as a needle is used to make a small hole in the alveolar mucosal tissues. Through this pinhole, special instruments are utilized to loosen the gingival tissues gently and slide the gingiva to cover the denuded root surface. Hereby, all the muscular and fibrous adhesions are released until the flap can freely move coronally without any tension. Since it only involves the adjustment of the position of the existing gingival tissues coronally, there are no incisions, no grafts, and no sutures [4]. But like any surgery, this technique has some limitations which could affect its success, such as medically compromised patients, heavy smokers and any medications that disturb the healing process or immunity response. Besides, occlusal discrepancies, parafunctional habits, bone defects, the amount of keratinized gingiva and gingival phenotype act as risk factors for PST achievement [5].

However, in this report, we used propolis as a natural anti-infective, anti-inflammatory, and anti-oxidant agent for root conditioning and to enhance faster healing. Propolis is a generic name for a complex mixture of resinous substances collected by honeybees from parts, buds, and exudates of plants. It contains a wide-ranging spectrum of chemical compounds that have many positive biological activities, including anti-oxidative and anti-inflammatory effects of polyphenols which inhibit the appearance of reactive oxygen species (ROS) in the inflammatory cascades [6]. Its antioxidant activity is even more potent than the antioxidant activity of vitamin C [7].

Also, propolis contains active compounds such as caffeic acid, caffeic phenyl ester, artepillin C, quercetin, resveratrol, galangin, and genistein which increase the antiseptic, antibacterial, antifungal, antiviral and immunomodulatory actions [6,8]. The activity of propolis against oral bacteria has been explored, suggesting the effectiveness of propolis as an anti-cariogenic product and anti-periodontal pathogens such as Porphyromonas gingivalis [9,10]. Besides, it acts as anti-plaque and anti-calculus agents where it decreases the formation of oral calcium-phosphate precipitate [11] by reducing the conversion of amorphous calcium phosphate to hydroxyapatite [8]. In addition, its antifungal efficacy against *Candida albicans* and its antiviral effects on the avian influenza virus were reported [12]. Moreover, its application along with antibiotics increases their efficacy by 10 to 100 times and seems to have a significant synergistic effect on them [12].

However, propolis shows an increase in extracellular matrix (ECM) components during the initial phase of wound repair, followed by a reduction in the ECM molecules and stimulation of the expression of transforming growth factor-β (TGF-β) [6] that participates in the early phases of wound repair such as hemostasis and inflammation [6]. Moreover, it was reported that propolis has an analgesic property similar to Aspirin but with minimal side effects [13].

Besides, it enhances protein synthesis, cell mitosis, increased cell metabolism and collagen synthesis, which are critical for gingival regeneration. According to our research, no previous studies using propolis as conditioning material in root coverage surgeries have been conducted, so we investigated in this case report, a novel protocol for the treatment of GR using PST with propolis application. This report is in line with SCARE 2020 guidelines detailed in the literature [14].

## 2 Case presentation

A 58-year-old healthy female patient was referred to the periodontal clinics, in our institution with the chief complaint of decreased gum height and appearance of part of roots related to upper left canine and premolar during smiling. These findings were started and noticed in the past 2 years, which affected her confidence and physiological state. In addition, the patient revealed a negative history of any medications or medical conditions. Her family and social history were insignificant. She didn't have any previous oral intervention for her complaint.

## 3 Clinical and periodontal examination

We observed that the patient had maintained relatively good oral hygiene where minimal amounts of plaque deposits were detected as the average plaque index was 0.9 giving evident faulty brushing habit. The patient disclosed that she used to brush her teeth by manually horizontal brushing technique two times/day without any auxiliary aids.

**Fig. 1:**
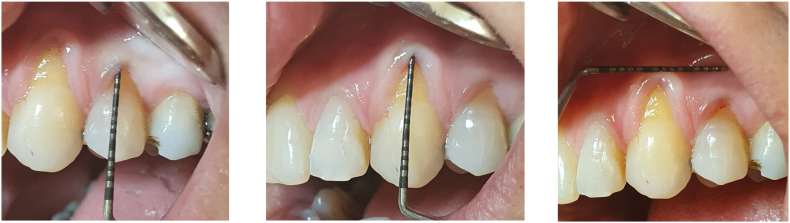
Preoperative pictures.

Periodontal measurements were done using UNC-15 periodontal probe (colour code at 4th, 9th, 14th mm), where the probing depth (PD) = 1 mm in the midfacial probing depth of both teeth, recession height (RH) = 4 mm in tooth # 23, while tooth #24 RH = 2 mm, with a width of keratinized tissues (WKT) = 3 mm. A rolling test was achieved using the periodontal probe in a horizontal position in an apico-coronal direction reaching the gingival margin of tooth #23, which confirmed that the gingival margin was at the mucogingival junction (MGJ). No proximal attachment loss, no bleeding on probing and no radiographic bone loss evidence were detected in the affected teeth. Only mild gingival inflammation with rolled marginal gingiva and blunted interdental papillae were observed (Fig. 1).

## 4 Treatment considerations

Affect teeth were recorded as gingival recession Millar's class II related to #23 and Class I related to #24. Although the patient's priority was esthetic concerns, she preferred not to have a secondary surgical site for the donor graft. Therefore, to cover the exposed root and increase the keratinized gingival zone with minimally invasive procedures, a pinhole technique was planned for her. The patient was shown visual pictures of this technique to explain the steps of the treatment plan. Additionally, the patient was well-informed about all instructions and complications. Written informed consent was obtained and the approval was achieved for the surgical procedure, photos and publication permission.

## 5 Surgical procedures

**Fig. 2:**
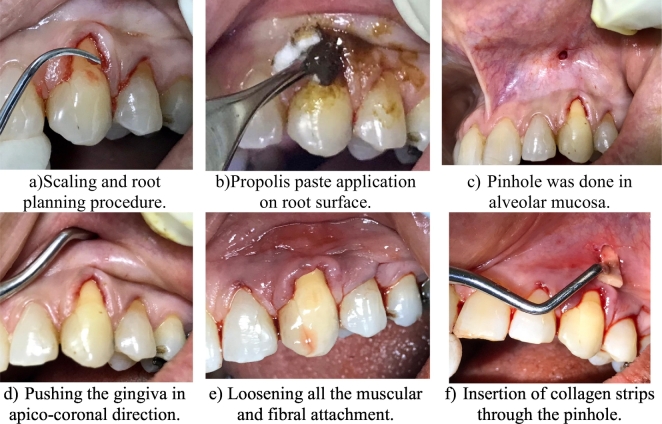
a–f: Pinhole surgical technique.

First, as a preliminary routine, scaling and root planning were done by the hygienist to prepare teeth for phase II therapy (Fig. 2). On the appointed day, the periodontist administrated the local anaesthesia infiltration and the roots were conditioned with propolis paste for 3 min using a cotton pellet as shown in Fig. 2. Then, the senior periodontist created a pinhole by piercing the alveolar mucosa 4 mm above the mucogingival junction (MGJ) apical to the recession defect as shown in Fig. 2. Following that, a curved Orban knife and tunneling instruments were used to loosen and expand the gingival tissues, pushing the gingiva downwards in an apico-coronal direction until it reached the desired position coronal the CEJ. Full-thickness splitting was established after the elevation of the periosteum and releasing all muscular and fibrous adhesion, creating free mobile gingiva (Fig. 2). Subsequently, a collagen membrane was cut into small strips and were inserted through the hole to the interdental papillae to assist and maintain the gingiva into its coronal position, as shown in Fig. 2. Gentle digital pressure was performed for 3 min to hold the gingiva in its new position and propolis was applied again on the gingiva postoperatively for 3 min.

**Fig. 3:**
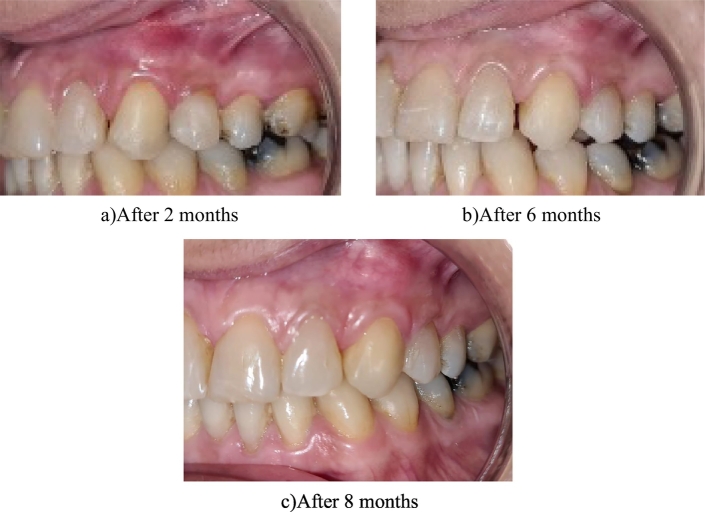
a,b,c: Postopertive pictures.

The patient was instructed to avoid any hot drinks for the first 24 h and to apply the propolis paste twice a day for one week, and not to brush the surgical area for 4 weeks. A postoperative analgesic was prescribed 3 times/day for 3 days. Chlorohexidine mouth rinse was prescribed after 10 days of surgery to avoid any physical disturbance. After 4 weeks, the patient was instructed to use an extra soft brush for brushing in vertical strokes, moving in a vestibular–coronal direction only. The photos were taken before, during surgery and at the follow-up periods until 8 months Fig. 3.

## 6 Clinical results

Immediately after PST, exposed roots were covered 100% without either incisions or flap reflection. There was no need for a graft or donor site. There were no postoperative complications except mild pain on the day of surgery. Neither gingival inflammation nor bleeding was observed.

Follow-up examinations revealed a stable position of the gingiva covering the roots with decreased GML (Fig. 3). Recession height (RH) and recession width (RW) were calibrated giving zero. Our results revealed that there was an increase in gingival volume and biotype thickness in both teeth #23 and # 24 as well as an increase in WKT of both affected teeth. The patient was highly satisfied by the esthetic outcomes that we achieved.

## 7 Discussion

In this case, we used PST to cover the exposed roots of #23 and #24. PST is a minimally invasive surgical technique that covers the exposed roots by repositioning the gingiva coronally without the use of a scalpel. In contrast to any other surgical flap, it doesn't need a donor surgical site, gives immediate esthetic results, consumes less time and covers any number of multiple roots [4].

In addition, we used propolis paste in root conditioning and postoperatively as it is a natural anti-infective and anti-oxidant agent to enhance the healing process. There was neither postoperative inflammation nor bleeding. This is possibly due to propolis's anti-inflammatory effect, which inhibits the lipoxygenase and cyclooxygenase enzymes, preventing the conversion of arachidonic acid to prostaglandins and leukotrienes [8], as well as the stimulation of expression of transforming growth factor-β (TGF-β) [6], which participates in the hemostasis during wound healing. These results correspond to Coutinho [10] who concluded that the use of propolis irrigation sub-gingivally decreased the periodontal pathogens, inflammation and bleeding even after 6 weeks of its application. Furthermore, Carvalho Magro [15] reported that propolis rinsing improved wound healing and displayed anti-inflammatory properties in the surgical dental sockets. Also, Olczyk et al. [16] discovered that propolis has high efficacy in the synthesis of collagen type I and III which in turn enhances the healing process and gives the gingiva the fibrotic appearance.

Furthermore, PST was less traumatic than other root coverage surgeries, resulting in minimal observed bleeding during the procedure, which increases the patient comfortability, visibility and speed the healing. This is because there was no actual separation of the underlying tissues, preserving the tissues' integrity without disrupting the vascular supply, which also explains the absence of healing complications or scar formation, which subsequently gives additional biological and esthetic advantages [17].

Furthermore, we used resorbable collagen strips to regenerate and support the periodontal tissues in their new position, which was done in many previous studies [4,18,19] which confirmed the regeneration of the tissues. However, more histological evidences are needed.

We measured the following parameters at baseline and after 8 months; recession height (RH) which was gauged from the CEJ to the gingival margin, recession width (RW) which was calibrated as the distance between the gingival margin from mesial to the distal end at the level of CEJ and width of keratinized tissue (WKT) which was measured as subtracting probing depth from the total of keratinized gingiva.

Our results revealed that there was an increase in gingival volume and biotype thickness in both teeth #23 and # 24, these outcomes were also supported by Chao [4]who concluded that PST is capable of increasing the tissue volume and give stable predictable results if the presented tissue thickness is 0.8–1 mm minimally. Furthermore, there was an increase in WKT in both teeth from baseline to 8 months, these results were also concluded by Anuroopa et al. [17], Reddy [18], Agarwal et al. [20] and Chao [4].

One of the most significant findings is that the patient was able to observe the results immediately, also PST gave excellent esthetic results with homogenous gingival colour in contrast to free gingival graft augmentation. Consequently, this increases the patient satisfaction outcomes. This result was corresponding to Kerner et al. [21] who concluded that the colour of the gingiva is the main goal of patient satisfaction and esthetic outcomes rather than the percentage of root coverage. In addition, perfect esthetic results and the colour match were also documented in other studies [4,18,20].

The patient reported postoperative pain on the day of surgery, this outcome was in agreement with Agarwal et al. [20]who reported that pain and bleeding for a short duration while Reddy [19] stated that there was pain, bleeding and swelling for the duration of two days postoperatively.

It is essential to be mentioned that there are limitations of the PST, including the technique sensitivity and the use of especial instruments to elevate the flap elevation without exposure of the inside tissue which increases the risk of flap proliferation, this was also mentioned as a limitation in case series done by Reddy [19] in 2017.

## 8 Conclusion

PST is a promising modality that reaches the periodontist ambition for gingival recession defects. Also, using propolis protocol in periodontal surgeries should be considered for root decontamination and enhancement of healing. However, more clinical and histological studies are highly recommended to be addressed and to compare different approaches of the pinhole technique for root coverage.

## Abbreviations

CEJ: Cementoenamel junction
GR: Gingival recession
MGJ: Mucogingival junction
PD: Probing depth
PST: Pinhole technique
RC: Root coverage
RH: Recession height
ROS: Reactive oxygen species
TGF: Transferring growth factor
WKT: Width of keratinized tissues

## Consent

A written informed consent was obtained from the patient for publication of this case report and accompanying images. A copy of the written consent is available for review by the Editor-in-Chief of this journal on request.

## Financial support and sponsorship

Nil.

## Ethical approval

IRB approval was obtained

Registration of research studies

Not applicable

Provenance and peer review

Not commissioned, externally peer-reviewed

## Patient consent

Informed consent and verbal approval were obtained from the patient after providing her with a full explanation before any treatment procedures.

## Declaration of competing interest

None.
